# Making A Difference: Launching a Multimodal, Resident-Run Social Emergency Medicine Program

**DOI:** 10.5811/westjem.18509

**Published:** 2024-08-16

**Authors:** Naomi P. Newton, Christopher Freeman, Patricia Panakos

**Affiliations:** * Emory University School of Medicine, Department of Emergency Medicine, Atlanta, Georgia; † Jackson Health System, Department of Emergency Medicine, Miami, Florida

## Abstract

**Introduction:**

Social medicine seeks to incorporate patients’ social contexts into their medical care. Emergency physicians are uniquely positioned to address social determinants of health (SDoH) on the frontlines of the healthcare system. Miami-Dade County (MDC) is a diverse and socially vulnerable area. In 2020, the University of Miami-Jackson Health System (UM-JHS) emergency medicine (EM) residency program launched a multimodal, resident-led Social EM program to identify and address SDoH in the emergency department (ED).

**Methods:**

We use a four-pillar approach to SDoH in the ED: Curriculum Integration; Community Outreach; Access to Care; and Social Justice. Residents graduate with a knowledge of Social EM principles through an 18-month curriculum, an elective, and a longitudinal track. We developed sustainable initiatives through interdepartmental and community-based partnerships, including a Narcan distribution initiative, an ED-based program linking uninsured patients to follow-up care, a human trafficking education initiative, and a quality improvement initiative for incarcerated patients.

**Results:**

Given that the 18-month curriculum was launched in 2022, a full rotation of the curriculum had not been completed as of this writing, and data collection and analysis is an ongoing process. The initial pretest and post-test survey data show improvement in knowledge and confidence in managing Social EM topics. The Narcan initiative has screened 1,188 patients, of whom 144 have received Narcan. The ED-based patient navigation program has enrolled 31 patients to date, 18 of whom obtained outpatient care. Analysis of the impact/effectiveness of the program’s other initiatives is ongoing.

**Conclusion:**

To our knowledge, this is one of the most robust social EM programs to date, as many other programs primarily focus on service opportunities. Rooted in the revised principles of Bloom’s taxonomy of cognitive learning, this program moves beyond understanding Social EM tenets to generating solutions to address SDoH in and outside the ED.

## BACKGROUND

Social medicine, or the incorporation of patients’ social contexts into their medical care, has become a vibrant, interdisciplinary movement that has gained traction in medical schools, residencies, and at the national level. Social medicine emphasizes the importance of social determinants of health (SDoH), or “the conditions in the environments where people are born, live, learn, work, play, worship, and age that affect a wide range of health, functioning, and quality-of-life outcomes and risks.”^1^ The US Department of Health and Human Services lists five core SDoH to consider during patient care: economic stability; education access and quality; healthcare access and quality; neighborhood and built environment; and social and community context.^1^

Although SDoH can be applied to all specialties, they are perhaps most relevant to emergency medicine (EM). Passage of the Emergency Medical Treatment and Labor Act (EMTALA) in 1986^2^ was acknowledgment that emergency physicians are often the only link to the healthcare system for patients with financial limitations. Emergency physicians are estimated to provide two-thirds of acute care for all uninsured patients and half of acute care for all Medicaid patients.^3^ Whether they are rushing a patient to CT, leading their team during a resuscitation, or evaluating patients in a crowded hallway, emergency physicians are immersed in longstanding, complex social issues: trauma; poverty; homelessness; mental health disorders; etc. Therefore, recognizing the effects of SDoH on patient care is critical in the ED.

Jackson Memorial Hospital (JMH) is the primary training site for the University of Miami-Jackson Health System (UM-JHS) EM residency program. It is also the third largest public hospital in the country. The UM-JH Social EM program was launched in 2020 to improve the incorporation of patients’ social contexts into their care.

### Needs Assessment

When designing a Social EM program, keeping the residency’s location and patient population in mind is important. Like most EDs across the nation, the JMH ED is a place of refuge for patients whose SDoH may prevent them from obtaining care elsewhere. As a safety-net hospital in the seventh most populous county in the nation,^4^ JMH serves a particularly diverse patient population with striking socioeconomic needs. The UM health system conducted formal needs assessments of Miami-Dade County (MDC) from 2019–2022, and the UM-JHS Social EM program was designed to reflect these needs.^4^ ^,^ ^5^

The UM-JHS Social EM program was designed to ensure that all residents graduate with the ability to incorporate their patients’ SDoH into ED care regardless of their ultimate practice locations. However, certain aspects of this program were designed to address the unique needs of MDC—a “minority-majority” community that experiences challenges with English proficiency, and in which 20% of the population lives below the poverty level.^4^ ^,^ ^5^

## PROGRAM GOALS

Bloom’s taxonomy of cognitive learning objectives outlines six levels in the cognitive domain: knowledge; comprehension; application; analysis; synthesis; and evaluation.^6^ Over time, scholars have sought to revise this framework and, when taken as a whole, these revisions place less emphasis on a linear progression through each level.^6^ Instead, there is an increased focus on generating new hypotheses and developing projects that use and expand upon the acquired knowledge.^6^ Therefore, the UM-JHS Social EM program seeks to shift its participants from purely understanding SDoH as they pertain to EM, to generating effective solutions for addressing these SDoH in and outside the ED. The Social EM program outlines six goals for its residents, who are then tasked with generating effective solutions and designing their own measurable outcomes for each goal. Upon successful completion of this program, residents should be able to:

1. Define and identify SDoH in the ED and apply these principles to bedside care.
2. Engage with MDC outside the ED and address its social and medical needs through longitudinal involvement in local outreach initiatives.
3. Solidify and share acquired knowledge through an 18-month, multimodal curriculum.
4. Identify and seek to address barriers to medical care experienced by patients who use the ED as their main source of healthcare.
5. Identify and seek to address recurrent social justice issues encountered in the ED.
6. Enact positive change through advocacy and quality improvement initiatives at hospital-wide, local, and/or national levels.

## PROGRAM PARTICIPATION

**Figure 1.**
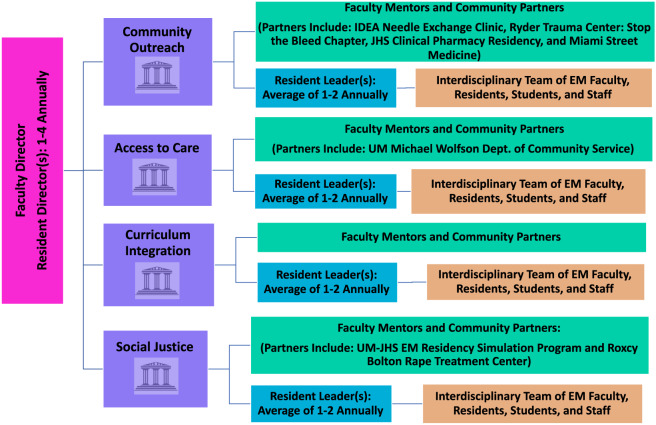
Social emergency medicine program organization and division of labor. Program directors consist of one faculty director and 1–4 resident directors (EM residents selected via a formal application process). Program directors oversee initiatives across all four pillars but spend additional time leading Curriculum Integration initiatives to ensure a seamless incorporation of Social EM principles into residency training. Pillar leaders are EM residents who are selected by program directors via a formal application process; they design and oversee initiatives in their assigned pillars. Faculty mentors are generally core faculty in the EM department with expertise in their assigned pillar. However, faculty in other specialties at UM-JHS may also serve as mentors if they currently oversee a community or hospital-based initiative that collaborates with the Social EM program. (For example, a faculty mentor from the family medicine department oversees the IDEA Needle Exchange Clinic.) Anyone affiliated with the EM department may serve as a team member. Team members work directly with their assigned resident leaders and divide the tasks required to launch and publicize initiatives.

Since its launch in 2020, the program has been divided into four pillars that address core areas within Social EM: Curriculum Integration; Community Outreach; Access to Care; and Social Justice (Figure 1). Initiatives within each pillar will be discussed in a separate section. Anyone affiliated with the UM-JHS ED can participate in initiatives across all four pillars. Many of these initiatives are longitudinal, allowing for varying levels of participation throughout residency. Additionally, this program also offers leadership, peer teaching, and scholarly opportunities that may count toward existing residency requirements.

The UM-JHS has a three-year EM residency program, and each of its classes (postgraduate years [PGY] 1–3) is comprised of 14–15 residents. EM residents are not required to participate in the Social EM program but are encouraged to do so. They may choose to serve as program leaders (Figure 1), participate in the longitudinal track and/or two-week elective (discussed in detail in subsequent sections below), or to participate in individual initiatives as their schedules allow. However, Social EM program leadership developed a formal curriculum to ensure that all residents graduate with a solid understanding of core Social EM principles, regardless of their level of involvement with the program; this will be discussed in a separate section.

## CORE LEADERSHIP HIERARCHY

This program was designed to be executed by residents in collaboration with faculty, medical students, and staff. The program was structured into a core leadership hierarchy to appropriately divide the labor of designing and launching initiatives that pertain to each pillar, while ensuring that residents complete their existing clinical and academic requirements (Figure 1). This leadership hierarchy organizes, executes, and publicizes the program and its initiatives.

### Directors

A faculty director and at least one resident director oversee the program together (Figure 1). The original directors, Patricia Panakos, MD, and Naomi Newton, MD, authored this paper and conceptualized the program together in Fall 2020. The collaboration between Drs. Panakos and Newton was borne from a shared passion for social medicine and a desire to implement an EM residency-based program to address the SDoH of patients in MDC. Dr. Panakos is the associate program director for the UM-JHS EM residency and has undergone formal training in curriculum development. She has also developed ED-based public health initiatives at JHS, such as a universal screening program for communicable diseases, including HIV, hepatitis C, and syphilis. Dr. Panakos continues her role as faculty director for the social EM program. Dr. Newton is an alumna of the UM-JHS EM residency and served as chief resident during her final year of training. She assumed the role of the social EM program’s resident director as a PGY-1 and transitioned her position upon her graduation in 2023. She has also collaborated with Dr. Panakos on public health initiatives, including a universal HIV screening initiative in JHS’s pediatric ED. Dr. Newton is currently pursuing a two-year fellowship in health policy and advocacy at Emory University.

Given that there was no precedence for such a program at UM-JHS, Drs. Panakos and Newton worked almost daily to create the program and maintain its sustainability, while also completing their existing clinical and academic responsibilities. Drs. Panakos and Newton designed the program’s overall structure, created a formal selection process for pillar leaders, and identified community partners and faculty mentors with expertise in Social EM. They presented a formal proposal that was approved by both the chair of the ED at JMH and the UM-JHS EM residency program director in October 2020. They also designed and launched the 18-month curriculum, two-week elective, and longitudinal track, which are described in subsequent sections of this paper. To account for continued program growth, the original directors selected four new resident directors for the 2023–2024 academic year via a formal application process (Figure 1).

Directors approve proposed initiatives across all pillars and work directly with pillar leaders to track progress and troubleshoot challenges. They check in remotely with pillar leaders at least bi-monthly to ensure timely project completion. They also promote the program at a departmental and hospital-wide level and help pillar leaders identify faculty and community partners (Figure 1). Resident directors are recognized with a Social EM leadership award upon their graduation.

### Resident Leaders

An average of two PGY-1 or PGY-2 EM residents lead each pillar. Interested residents apply for this position via a brief electronic application (Google Survey) at the start of the academic year and are selected by the directors. Residents generally do not serve as leaders of more than one pillar, as this position must be balanced with existing residency obligations. Resident leaders report directly to the directors and dedicate an average of two to four hours per week to their roles. As leaders progress through training, they may either remain in their leadership roles or transition their roles to incoming PGY-1s and PGY-2s. All resident leaders who have served for at least one year are recognized with a special award upon graduation from residency.

Leaders focus on designing initiatives that pertain to the goals of their assigned pillar. They identify appropriate partners within JHS and MDC to aid in developing and launching these initiatives (Figure 1). Partners include JHS faculty (including those in non-EM specialties), local outreach organizations (many of which already had established relationships with UM-JHS through medical student involvement), and other JHS-affiliated residency programs (eg, pediatrics, internal medicine, family medicine). Interdisciplinary collaboration prevents the Social EM program from “re-inventing the wheel” and helps initiatives achieve success with fewer funding, resource, and logistical restrictions. Resident leaders delegate day-to-day tasks to an interdisciplinary team to divide the labor of executing these initiatives. Leaders are required to check in remotely with their team members at least monthly to discuss progress on pillar initiatives.

### Interdisciplinary Teams

Team members divide the tasks required to launch initiatives within their assigned pillar. They are required to dedicate a minimum of one to two hours per week on these tasks and check in regularly with their pillar leaders as previously discussed. Those who desire to do so may participate in more than one pillar team. Participation in a pillar team is open to anyone in the UM-JHS ED. However, during the first three years of the program, teams were primarily comprised of EM-bound UM medical and pharmacy students, JHS clinical pharmacy residents, and hospital staff (eg, nurses and social workers).

## PROGRAM DESIGN: THE 4-PILLAR APPROACH

In the following section, we provide a broad overview of each pillar’s objectives and highlight several key initiatives within each pillar. When relevant, please see the corresponding appendices for additional details.

### Curriculum Integration

This pillar incorporates the tenets of Social EM into residency training to empower future generations of emergency physicians to apply Social EM principles to their care. This is the only pillar that requires all EM residents to participate because its initiatives have been incorporated into the existing residency curriculum. Doing so ensures that all EM residents graduate with an understanding of SDoH and the principles of Social EM, regardless of their level of involvement in other pillars. Of note, approval from the institutional review board was not required for the development of this curriculum.

We developed and launched a multimodal, 18-month Social EM curriculum that has been incorporated into the existing 18-month residency didactic schedule (Appendix A). The curriculum covers 18 core social EM topics (Table 1) and includes journal clubs, simulation cases, lectures, problem-based learning, and interactive group discussions. The curriculum is led by faculty and residents with expertise or interest in the core topics. Social EM leadership assists presenters in identifying learning objectives for each session, selecting topics, and developing content. All conference attendees participate in pre- and post-surveys to assess their baseline knowledge and the effectiveness of each didactic session. Residents are also asked to evaluate the Social EM curriculum during the annual residency program evaluation. Surveys and results are discussed further in the Impact/Effectiveness section of this manuscript.

**Table 1.** 18 core areas of study were chosen to be covered monthly during the 18-month Social EM curriculum. This curriculum is integrated into standard residency didactic schedule, which repeats every 18 months. Using a multimodal learning format, topics can be presented as traditional lectures, case-based discussions and journal clubs (“Cases”), or simulations. The initial modalities for each topic are listed below; the modalities used for each topic will change every 18 months (eg, the pediatric health lecture would be presented as either a case or simulation 18 months later). Additional details regarding logistics and implementation can be found in Appendix A.

Lectures	Cases	Simulations
1. Social determinants of health	7. Implicit bias/racism	13. Human trafficking and domestic violence
2. Healthcare coverage and access	8. Homelessness	14. Substance abuse and harm reduction
3. Financial stability	9. Health literacy	15. Caring for incarcerated patients
4. Frequent ED utilizers	10. Immigration	16. Highly communicable diseases/STI epidemics
5. Women’s health	11. Resource insecurity	17. Language and cultural barriers to healthcare
6. Pediatric health	12. Trauma-informed care	18. Gender identity

*ED*, emergency department; *STI*, sexually transmitted infection.

In 2022, we launched the two-week Social EM elective for residents who desire a more in-depth experience with the program (Appendix B). This elective is open to PGY-2 EM residents during their elective block and is comprised of service opportunities, self-directed study, peer teaching, and initiative participation across all pillars. The PGY-2 rotation schedule is designed so that only one resident completes an elective in any given month. Therefore, the experience is personalized for each participating resident. Social EM directors work with the resident ahead of time to design an elective schedule that ensures participation across all pillars but allows them to engage more deeply within their pillar(s) of interest (Appendix B).

**Figure 2.**
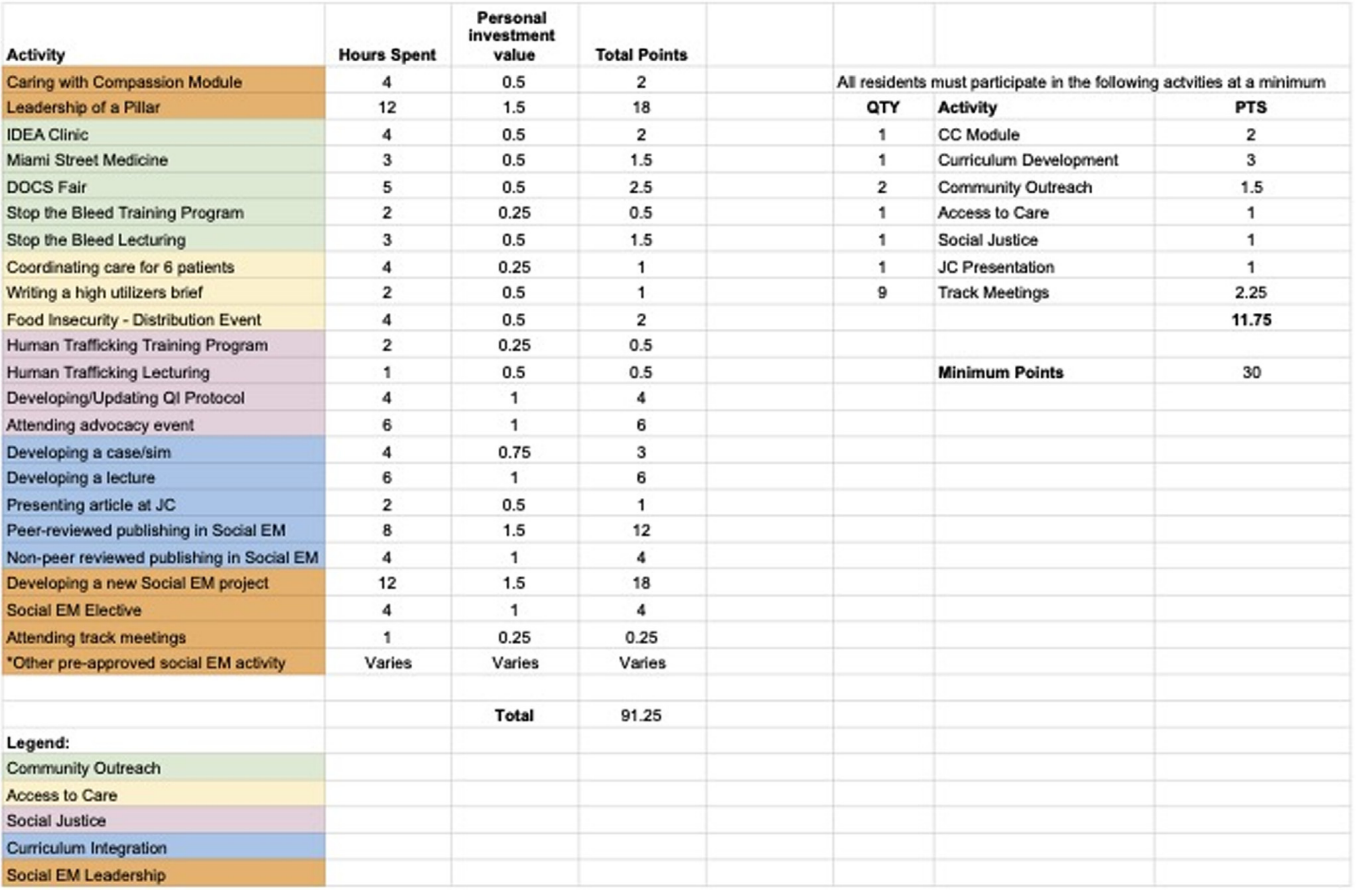
Point system for the 18-month longitudinal track. Note that opportunities in each pillar may vary over time. This figure lists opportunities from the fall of 2023.

We also designed a longitudinal track that was launched the 2023–2024 academic year. Track participants engage in a set number of outreach events, quality improvement initiatives, peer teaching, and self-directed study over 18 months. The requirements are based on a point system that ensures engagement with all pillars but allows for deeper exploration in areas of individual interest. Residents must reach a minimum of 30 points to complete the track (Figure 2). Requirements include a longitudinal scholarly activity that culminates in a presentation at the local, regional, or national levels (eg, developing a project to address food insecurity). They must also participate in the Social EM didactic curriculum through peer teaching, developing new elements to the curriculum, and mentoring medical students. Participants log their progress via an online form and must attend a minimum of nine monthly track meetings with the Social EM directors over an 18-month period. Upon graduation, residents who complete the track will receive a Distinction in Social EM.

### Community Outreach

This pillar was designed to establish a meaningful presence in MDC beyond bedside care and to address social issues through partnerships with local organizations. For example, through a partnership with Miami Street Medicine, participants join an interdisciplinary team in providing regular medical care at locations commonly occupied by Miami’s homeless population. Through a partnership with the Stop the Bleed Campaign, participants undergo formal training to serve as local instructors. Participants then lead workshops that teach non-medical community members to perform bystander cardiopulmonary resuscitation and stabilize victims of violence until first responders arrive. Participants may serve as instructors for Stop the Bleed events throughout MDC, as their schedules allow.

This pillar launched a Narcan program in July 2022, in partnership with the UM IDEA (Infectious Disease Elimination Act) Needle Exchange Clinic and the UM Michael Wolfson Department of Community Service (DOCS). This program seeks to address South Florida’s opioid epidemic and is in keeping with the statewide Emergency Treatment for Suspected Opioid Overdose Act.^7^ At community health fairs, participants provide free opioid use disorder (OUD) screening, based on Diagnostic and Statistical Manual of Mental Disorders, 5^th^ ed, criteria.^8^ Narcan is subsequently distributed to those identified to be at high risk for life-threatening overdoses, and additional OUD counseling and training on safely administering Narcan are provided.^9^

### Access to Care

This pillar links chronically ill patients, high ED utilizers, and the uninsured to outpatient care. It also seeks to centralize existing social support resources within UM-JHS and efficiently address SDoH at the bedside. Through a partnership with DOCS, uninsured patients presenting to the ED with chronic complaints are paired with long-term patient navigators, who help them secure affordable outpatient care upon discharge.

The High Utilizers Initiative aims to streamline the care of patients who frequently visit the ED. Participants conduct chart reviews of individuals flagged as frequent utilizers in the electronic health record and create patient care briefs that auto-populate in their charts. These patients often present to the ED numerous times a week and receive care from different clinicians each time. By consolidating their pertinent medical information, these briefs allow for better, more streamlined patient care with less repetition of tests and procedures. The briefs also lessen the cognitive load of the clinician, decreasing the time spent on chart reviews and helping guide future care.

Many patients present to the ED with conditions that are exacerbated by a lack of basic resources. It is challenging to address these complex SDoH amidst the time constraints of ED care, and EDs can no longer rely heavily on social workers for assistance, due to the nationwide social worker shortage.^10^ This pillar partnered with Miami Street Medicine and the JMH Pharmacy Department to create resource guides for patients and clinicians in response to this need. Community resource guides (in English, Spanish, and Haitian Creole) provide information for affordable outpatient clinics, prescriptions, mental health services, temporary housing, meal programs, and substance use treatment centers, as well as resources for pregnant patients and victims of domestic violence. Clinician resources include referral information for resident-run subspecialty clinics, instructions for initiating buprenorphine in the ED and referring patients to medication-assisted treatment clinics, and algorithms for human trafficking screening.

### Social Justice

This pillar tackles health inequity and injustice issues through interdisciplinary education and quality improvement initiatives. The Human Trafficking Education Ambassador program, in partnership with JMH’s Rape Treatment Center, teaches clinicians to screen for and treat victims of human trafficking. Florida has the third highest number of human trafficking cases in the nation, and MDC is, sadly, a known trafficking hub.^11^ Trained residents lead interactive seminars, sharing HIPAA-compliant trafficking cases and teaching clinicians to identify and address red flags for trafficking.

This pillar also seeks to improve care for incarcerated patients in the ED, particularly concerning patient privacy and examinations in the presence of law enforcement. Initiatives include a recently published review on the barriers to caring for this population and recommendations to improve their delivery of care.^12^ We also implemented a simulation session on caring for incarcerated patients into residency didactics.

## IMPACT/EFFECTIVENESS

### Curriculum Integration

Social EM leadership is in the process of completing a formal impact assessment of the curriculum integration pillar of the program via a single-group, pretest-posttest design.^6^ Brief pre- and post-didactic session surveys are designed for each Social EM topic in the 18-month curriculum. Surveys are designed to assess baseline knowledge of the topic and the changes in this baseline knowledge after the session. Survey questions also address relevant epidemiological statistics and useful community resources for addressing the topic in MDC. Each post-survey ends with a blank section for participants to write in any additional feedback, which Social EM program leadership uses for subsequent didactic sessions.

For convenience, these surveys are administered via electronic forms; conference attendees scan QR codes to the forms before and after the session. All residents, faculty, students, and staff in attendance are eligible for participation in the surveys. However, thus far, survey participation has generally been limited to resident attendees, as faculty, staff, and student attendance is less consistent. Hospital badge numbers are used to compare individuals’ changes in pre- and post-session responses.

**Figure 3.**
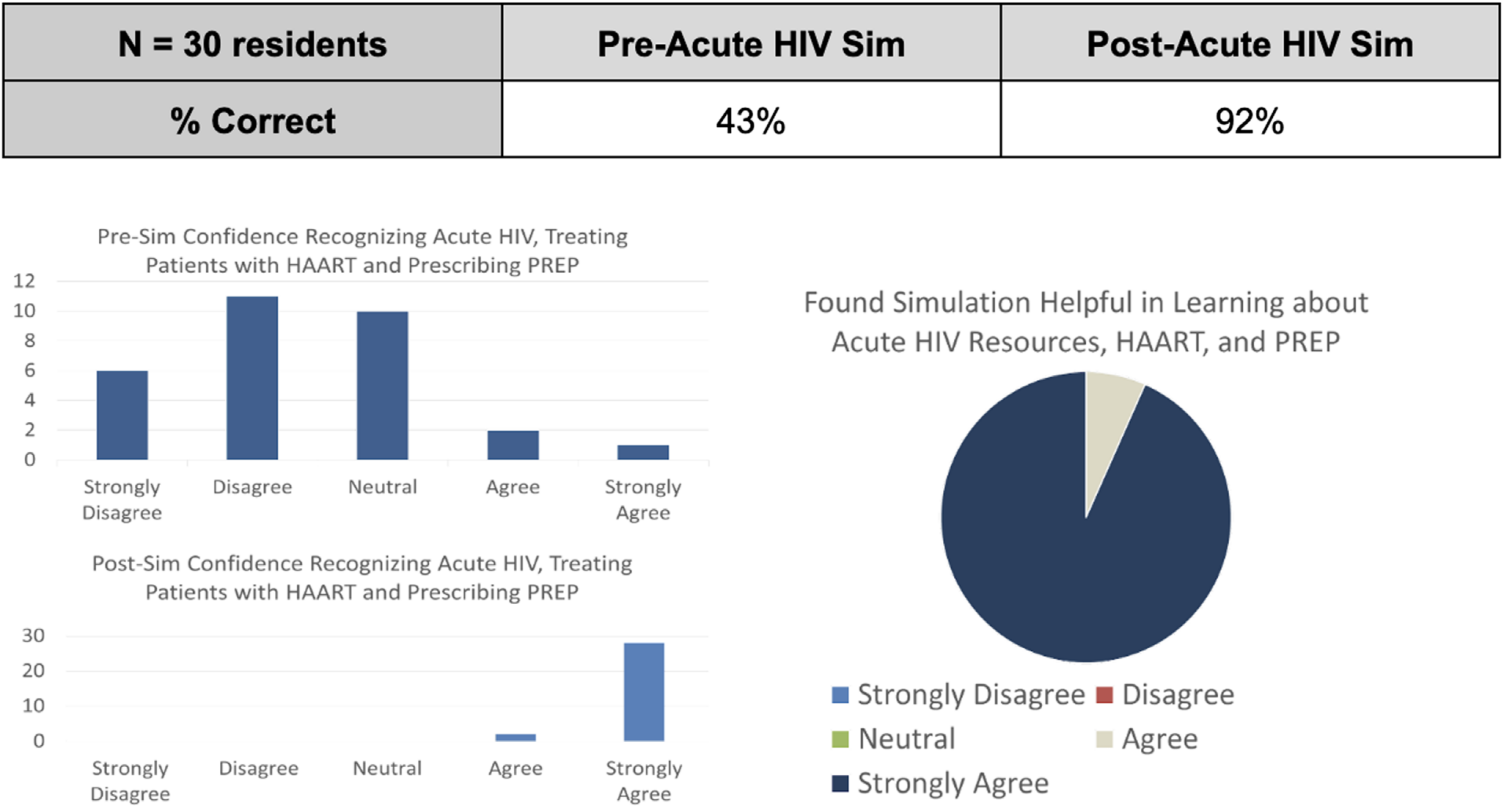
Comparison of key pre- and post-survey results after a simulation session on highly communicable diseases/STI epidemics; 30 residents completed the surveys. After the session, residents indicated an increased confidence in their ability to recognize acute HIV and initiate Highly Active Antiretroviral Therapy (HAART) or Pre-Exposure Prophylaxis (PrEP) treatment when indicated. Most residents found the session beneficial in learning about community resources for ED patients with HIV, as well as prescribing HAART and PrEP.

Since the 18-month curriculum was launched in 2022, a full rotation of the curriculum has not been completed as of this writing, and data collection and analysis is ongoing. However, thus far, the curriculum topics have been well-received, with residents indicating an improved confidence in their ability to recognize and address these Social EM issues at the bedside. For example, Figure 3 shows key results from the pre- and post-surveys administered during the first session of the formal curriculum in 2022—a simulation session on highly communicable diseases/sexually-transmitted infection (STI) epidemics (Table 1). These results suggest efficacy in improving baseline knowledge and confidence with the topic of acute HIV in the ED, including epidemiology, community resources, and initiating either highly active antiretroviral therapy or pre-exposure prophylaxis when indicated.

An impact assessment of the two-week elective is pending, as only one PGY-2 resident had completed at the time of this manuscript’s development.

### Community Outreach

It is challenging to concretely assess the impact of the Community Outreach pillar, as its service-driven initiatives are generally qualitative in nature. However, initial data from the Narcan Initiative highlights its impact on MDC. As of May 2023, the program screened 1,188 patients across MDC, of whom 144 received Narcan. In recognition of the Narcan Initiative’s current impact and continued growth, JMH’s Department of Emergency Medicine received the 2023 University of Miami Mitchell Wolfson Sr. Department of Community Service award.

### Access to Care

We are currently in the early stages of data collection to analyze the success of the Access to Care initiatives. Thus far, the patient navigation program has enrolled 31 ED patients. Of these patients, 18 were able to successfully complete their navigation goals and obtain outpatient care. This program has particularly benefitted non-English-speaking patients, whose language barriers can hinder their ability to navigate a complex system. For example, navigators were able to link a Spanish-speaking patient to outpatient oncologic care for her untreated gynecologic cancer. Recently, a homeless, uninsured patient living at the Miami Rescue Mission (MRM) was treated for an acute ulcerative colitis flare in the ED. After he was discharged, the navigators ensured that he obtained timely follow-up at an MRM-affiliated gastroenterology clinic, a student-run clinic staffed by UM faculty. We are continuing to publicize this program and encourage emergency clinicians to enroll their patients during their shifts. We are still in the data collection phase of the High Utilizers initiative.

### Social Justice

The Social Justice pillar initiatives experienced several launch delays due to COVID-19 pandemic restrictions and faculty turnover. Initiatives were officially launched in the 2022–2023 academic year, and data regarding their impact and effectiveness is pending. Thus far, human trafficking education ambassadors have given well-received lectures to JHS-affiliated clinics and to JMH’s family medicine, pediatrics, and internal medicine residencies.

### Overall Program Feedback and Support

Since the program is under the direct guidance of a current residency associate program director, there is continual communication between Social EM directors and EM residency leadership. Residency leadership actively engages with and provides insights into pillar initiatives, leading to timely changes to the program when deemed necessary. For example, previous feedback led to the development of the elective and longitudinal tracks. Residents in the core Social EM leadership team also obtain regular qualitative feedback from their peers and share this feedback with the Social EM directors. This program is also reviewed during the annual residency program evaluation committee meeting. This program has full EM departmental support.

### Additional Recognition/Awards

Overall, this robust, multimodal, resident-led Social EM program has rapidly grown over the last three years, despite the COVID-19 pandemic. In 2023, six of the 14 PGY-3 residents graduated with a Social EM distinction. The program’s interdisciplinary nature ensured its success, as multiple initiatives were launched without significant funding or administrative restrictions. The program is receiving increasing recognition. In addition to the previously mentioned community service award for the Narcan Initiative, the MDC chapter of the Stop the Bleed Campaign received a 2021 award from the mayor for its education initiatives in local high schools. In 2023, we were also honored to receive the 2023 ACEP Social EM Section Distinguished Program Award.

## LIMITATIONS

Residents’ availability often limits consistent participation in Social EM. Residents have multiple clinical and academic responsibilities, and as they progress through training, their time is further limited by searching for jobs and applying for fellowships. In response to this limitation, the elective and longitudinal track were developed to allow for flexible but regular participation, as many requirements can be completed during lighter rotations. The didactic curriculum also ensures that all residents will graduate with the same baseline knowledge of Social EM tenets. Additionally, the Social EM leadership will transition every two years, allowing junior residents with leadership roles to pass on their duties to incoming residents as they become senior residents.

Certain aspects of this program were designed to address some of the social issues that are particularly prevalent in MDC and may not be generalizable to other EM residency programs in the United States. Other residency programs seeking to develop their own Social EM initiatives should consider the unique needs of their patient populations when doing so.

The program’s first three years were dedicated to overall development, garnering participants, finding community partners, and launching initiatives in each pillar. Therefore, data collection to formally assess the program’s impact and effectiveness is still in process and is currently limited to initial data (unblinded pre- and post-tests completed by resident participants) from the launch of the 18-month didactic curriculum. This data may also be subject to selection bias, as most residents, faculty, students, and staff are excited about the Social EM program and want it to succeed.

## CONCLUSION

The University of Miami-Jackson Health System Social EM program was launched in 2020 to address the SDoH of patients in Miami-Dade County—an area of significant medical and social need. It targets critical social issues through four pillars: Curriculum Integration; Community Outreach; Access to Care; and Social Justice. This multimodal, resident-run program achieved rapid success in three years by developing sustainable initiatives in partnership with local organizations and other UM-JHS departments. Rather than focusing solely on service opportunities, this program enhances residents’ knowledge of SDoH, fosters the development of quality improvement initiatives, and provides opportunities to create meaningful change in the ED and the community. This program also provides residents with leadership and scholarly opportunities. We hope that this article will inspire other residencies to develop similar programs.

## Supplementary Information



