# Supplementary material for: Making A Difference: Launching a Multimodal, Resident-Run Social Emergency Medicine Program
Source: West J Emerg Med. 2024 Aug 16;25(5):739–47. doi: 10.5811/westjem.18509 (PMC11418877; doi:10.5811/westjem.18509)
Supplement: Supplementary file 1 [file wjem-25-739-s001.docx]

**Appendix A: CURRICULUM INTEGRATION PILLAR**

18-Month Social EM Curriculum:

The standard didactic curriculum for a 3-year EM residency program repeats every 18 months, to ensure that all core organ systems and areas of EM are covered twice by the time of graduation. Didactic sessions generally occur with all 3 residency classes together (except for some unique class-specific didactics) and are designed to benefit all learners, regardless of their stage of training.

The Social EM Curriculum was designed to be seamlessly incorporated into the existing didactic curriculum. This ensures that Social EM principles become core curricular content for EM residents and that all EM residents are repeatedly exposed to these principles throughout their training. The multimodal component of the curriculum allows learners to engage with this content in different forms every 18-months.

Ultimately, the goal is to incorporate Social EM topics into all existing lectures to demonstrate the effects of SDOH on all organ systems/pathology within the scope of EM. However, this 18-topic framework is a starting point, allowing for systematic integration of SDOH into the curriculum and assessment of its impact/effectiveness on learners.

**Logistics:**

- 18 Core Topics were selected by Social EM program directors (Table 1).
- Topics may be presented in a stand-alone manner (e.g., a lecture on SDOH) or may be integrated into an existing lecture (e.g., a simulation case in which key examination findings are not revealed unless the examiner removes an incarcerated patient from their shackles).
- One topic is covered during a mandatory residency conference session each month.
  - In the UM-JMH EM residency program, residency conference sessions include weekly morning didactics, quarterly “evening conference” sessions, monthly sessions in the simulation lab, and quarterly journal club meetings. Social EM topics can be incorporated into any of these conference sessions, allowing for scheduling flexibility.
- Topics are presented in a multimodal fashion: lectures, cases, or simulations (Table 1).
  - The modalities utilized for each topic may change every 18 months.
- Social EM program directors review all lectures, cases, and simulations in advance and help presenters design learning objectives for each topic. Simulation scripts are developed under the guidance of the UM-JHS Simulation Director.
- Tying the Social EM topics to the core clinical content covered in a given month (e.g., discussing Women’s Health during the OBGYN unit of the curriculum) is encouraged. This was not always feasible when the Social EM curriculum was first launched, as the didactic schedule had already been generated one year in advance.

**Who Presents the Material?**

- Guest speakers (faculty in and outside of the EM department who have expertise or interest in one of the topics).
- Residents:
  - Work with program directors and pillar leaders to create the content. Presentation dates are assigned based on the residents’ shift schedules.
  - Open to Social EM team leaders, residents, participating in the elective/ track, or any other residents interested in medical education or Social EM opportunities.
- EM-bound Medical Students:
  - As per UM-JHS EM residency policy, any interested medical student can attend residency conferences as their schedules allow.
  - Work with program directors and pillar leaders to create the content and practice their presentations ahead of time.

**Appendix B: SOCIAL EM ELECTIVE 2022-2023 FRAMEWORK**

*Note: This framework was used to design personalized Social EM elective experiences for PGY-2 residents in the 2022-2023 academic year. Several components of this elective have been adjusted since the authorship of this manuscript.*

Goals & Objectives:

1. Develop a deeper understanding of the social determinants of health, particularly in the context of emergency medicine.
2. Seek to address our patients’ social contexts on a deeper level, both in the emergency room and in the Miami-Dade Community.
3. Residents will have the opportunity to pursue a Focused Approach or a Broad Approach to the elective, based on their interests. Both approaches ensure exposure across all pillars, but the Focused Approach also allows the resident to develop a particular niche within the realm of Social EM.
   1. Options are described in detail below. The schedule is tailored to each resident, and the resident will work with the Social EM directors to create a final schedule/list of requirements.
4. All residents must complete The “Caring with Compassion” Learning Modules and Game, to ensure that a baseline understanding of the social determinants of health has been achieved by the end of the elective: https://caringwithcompassion.org/. (They will send a screenshot, indicating module completion to the faculty supervisor.)
5. Completing the Social EM Elective will count towards the Social EM Longitudinal Track.

Focused Approach:

In addition to sampling experiences within all 4 Social EM pillars, residents will pick 1-2 pillars (Community Outreach, Access to Care, Curriculum Integration, or Social Justice) to focus on during the 2-week elective. (They will spend more time on experiences in the pillar(s) of interest.) Residents will select options for involvement (varies each month). Involvement can be tailored to the resident’s interest. New ideas for projects/volunteer opportunities are also welcome if they can be completed in the 2-week period. The resident must participate in a minimum of 2 activities in their pillar(s) of focus, and a minimum of 1 activity in all other pillars.

Broad Approach:

An equal sampling of experiences within all 4 of the Social EM branches: Community Outreach, Access to Care, Curriculum Integration, and Social Justice. Residents will select options for involvement (varies each month). Involvement can be tailored to the resident’s interest. New ideas for projects/volunteer opportunities are also welcome if they can be completed in the 2-week period. The resident must complete a minimum of 1 activity in each of the pillars.

Monthly Options for Involvement- Sample from January 2023:

**Community Outreach:**

- IDEA Clinic: Volunteer on both Thursdays of the rotation.
- Miami Street Medicine: Spend 1-2 days rounding with the team depending on availability during the elective.
- DOCS Narcan Project: Volunteer at the Narcan Station if a Health Fair falls within the rotation’s time frame.
- Stop the Bleed: Complete a short training session (through Ryder Trauma Center) and subsequently lead one community teaching event during the elective.

**Access to Care:**

- High ED Utilizers Project:
  - Write 2 patient briefs (supervised by the project’s faculty mentor).
- Involvement in other projects, based on current needs, as listed below. (These opportunities may be subject to change):
  - - Social EM Resources for ED Patients/Providers:
      - Create binders w/ laminated patient & provider resources (in designated tabs) for each ED. Work with hospital administration to ensure that patient hand-outs are replaced monthly. Draw from resources previously added to the electronic health record; add handouts as needed.
      - Help promote use of resources in the ED.
- Access to Care Project:
  - Reach out to patients enrolled during your 2 weeks and work with the patient navigators to schedule outpatient follow up.

**Social Justice:**

- Human Trafficking Education Ambassadors:
  - Complete a brief (hour-long) “train-the-trainer” session (given by one of the residents in the Social Justice team).
  - Subsequently give a presentation on trafficking screening in the healthcare setting, as scheduled. (We have a running list of residency programs that are interested in these lectures. There are also opportunities for lecturing at other hospitals/clinics, or to other hospital staff, such as nursing staff.)
- Roxcy Bolton Rape Treatment Center:
  - Shadowing Sexual Assault Nurse Examiner (SANE) exams/cases.
  - Resident will be on-call from Monday through Wednesday during one week of the rotation. They will be contacted by the SANE nurse practitioner each time there is a new case. Resident must participate in a minimum of 2 cases during this on-call period.

**Curriculum Integration:**

- Write 1 lecture/case/simulation of your choice, based on available topics in the 18-month curriculum. Subsequently lead this didactic session and participate in data collection from the session.
